# Editorial: Diabetes, Hypertension and Cardiovascular Diseases

**DOI:** 10.3389/fphys.2021.765767

**Published:** 2021-10-22

**Authors:** Soo-Kyoung Choi

**Affiliations:** Department of Physiology, Yonsei University College of Medicine, Seoul, South Korea

**Keywords:** diabetes, hypertension, type 2 diabetes, cardiovascular diseases, cardiovascular dysfunction

Hypertension and diabetes are common comorbidities and associated with risk of life-threatening cardiovascular diseases (CVD). The prevalence of type 2 diabetes (T2D) continues to increase worldwide. According to World Health Organization (WHO), the number of cases of T2D has risen from 108 million in 1980 to 422 million in 2014 and is expected to grow to 642 million by 2040. Hypertension is more common, rising in prevalence with a recent estimate of 1.39 billion cases. Diabetes and hypertension are both associated with microvascular and macrovascular diseases and closely related due to similar risk factors such as endothelial dysfunction, vascular inflammation, arterial remodeling, atherosclerosis, dyslipidemia, and obesity. The aim of this special issue was to provide novel perspectives to the pathophysiological features of cardiovascular complications associated with diabetes and hypertension. We encouraged researchers to submit the articles approaching the topic in basic, translational and clinical aspects. Indeed, this special Research Topic collates 13 articles (10 original research articles, 2 review articles, and 1 opinion) showing the relevance between cardiovascular diseases, diabetes and hypertension in various perspectives.

The topic includes two articles that deal with pathophysiology in different animal model of diabetes (genetic and chemically induced model) which are streptozotocin (STZ)—induced diabetic mice and db^−^/db^−^ mice. The original article by Guerrero et al. showed that STZ-induced diabetic mice had higher urinary α-ketoglutarate (α-KG) and pro-renin receptor (PRR) expression along with augmented urinary angiotensin II (AngII) level and Na^+^ retention. They found that STZ-induced diabetic mice treated with montelukast (ML), receptor of α-KG (OXGR1) antagonist, did not show all these effects. They also reported that primary cultured inner medullary collecting duct (CD) cells treated with high glucose (HG) showed increased PRR expression, while OXGR1 antagonist prevented this effect. Their findings suggest this signaling pathway contributes to intratubular generation of AngII impacting on Na^2+^ handling.

Diabetic cardiomyopathy is characterized by structural and functional alterations of the heart. In this special issue, Pappritz et al. reported that CD362^+^ mesenchymal stromal cell (MSC) application decreased cardiomyocyte stiffness and increased arteriole density, which correlated with increased myocardial nitric oxide (NO) and cyclic guanosine monophosphate (cGMP) levels in leptin receptor deficient db^−^/db^−^ mice. However, the degree in improvement of cardiomyocyte stiffness following CD362^+^ MSC application was insufficient to improve diastolic function. These findings could contribute to understand the mechanism of diabetic cardiomyopathy.

Hypertension is one of the most important risk factors in heart failure. Pressure overload-induced cardiac hypertrophy is one of the main characteristics of cardiac remodeling which contributes to development of heart failure. The transverse aortic constriction model is well-known disease model to study pressure overload-induced cardiac hypertrophy and heart failure. In the present topic, Deng et al. showed mice subjected to 27G transverse aortic constriction (TAC) had severe cardiac dysfunction, severe cardiac fibrosis, and exhibited characteristics of heart failure at 4 weeks post-TAC. Compared with 27G TAC mice, 26G TAC mice showed a much milder response in cardiac dysfunction and cardiac fibrosis, and a very small fraction of the 26G TAC group exhibited characteristics of heart failure. They concluded that different degrees of TAC induce distinct phenotypes in C57BL/6N mice. Their results will provide important reference value especially for researchers who conduct pressure overload–induced cardiac hypertrophy studies using C57BL/6N mice or genetically modified mice with a C57BL/6N background. Endothelial dysfunction is closely associated with diabetes and hypertension. Thus, understanding the mechanism of endothelial dysfunction could contribute to identifying the targets for these diseases. In the special issue, Osman et al. reported that endoplasmic reticulum (ER) stress-generated microparticles (MPs) impaired angiogenic capacity of human umbilical endothelial cells (HUVECs) and reduced nitric oxide (NO) release, indicating an impaired endothelial function. These findings are clinically relevant as they will help in the process of identifying new therapeutic targets against MPs produced in conditions characterized by the activation of ER stress such as diabetes, obesity and metabolic syndrome.

On the other hand, Cho et al. reported the role of thromboxane A2 receptor-mediated signaling in the pathophysiology of pulmonary arterial hypertension (PAH) in this special issue. They showed the attenuation of nitric oxide (NO)/ cyclic guanosine mono phosphate (cGMP) signaling and the upregulation of (ROCK2) increase the sensitivity to thromboxane A2 (TXA2) in the pulmonary arterial hypertension (PAH) animal, which might have pathophysiological implications in patients with PAH. Their findings provide valuable information to understand the mechanism of pulmonary hypertension.

In this special issue, we tried to show various studies in different point of view regarding the topic, we even showed gestational diabetes mellitus (GDM) and hypertensive disorders (HDP) in HIV infected pregnant women. In the review article, Phoswa described that HIV/AIDS is associated with the pathophysiology of gestational diabetes and hypertensive disorders in of pregnancy. The highly active antiretroviral therapy (HAART) usage increased risk of gestational diabetes in HIV infected pregnancies due to diabetogenic effect by causing dysregulation of progesterone and prolactin and predispose HIV infected women to GDM. This review article helps on improving therapeutic management and understanding of the pathophysiology of GDM and HDP in the absence as well as in the presence of HIV infection by reviewing studies reporting on these mechanisms.

In this special issue, we introduced articles provide various statistical information about risk and prevalence of hypertension. Zhang, et al. reported that lower birth weight is nonlinearly correlated with higher risk of hypertension, and birth weight between 3.43 and 3.80 kg might represent an intervention threshold. Moreover, lower birth weight may interact with adult obesity to significantly increase hypertension risk. Liu et al. reported subclinical atherosclerosis (SCA) was highly prevalent in the hypertension population and the thoracic aorta was the most frequently affected vascular site. Elevated serum uric acid (SUA) concentration was significantly associated with the prevalence and severity of SCA regardless of territories. Ye et al. described the prevalence and associated factors of hypertension and phenotypes in Chinese children and adolescents during 1991–2015. They reported that from 1991 to 2015, the age-standardized prevalence of hypertension and phenotypes in Chinese children and adolescents increased dramatically. Sex and age disparities were found in the prevalence of childhood hypertension and phenotypes. For adolescents aged 13–17 years, general obesity and central obesity were positively associated with hypertension, whereas the South region was a negatively related factor. For diastolic hypertension (IDH), sex, age, location (urban or rural), region (north or south), body mass index (BMI), waist circumference (WC), general obesity, and central obesity were associated factors. Older age group, general obesity and central obesity were related to the increased prevalence of isolated systolic hypertension (ISH), and only general obesity associate with the elevated prevalence of IDH. It provides novel insights into several aspects of the national challenge of childhood hypertension in China. On the other hand, Lin et al. determined that whether long-term intensity of glycemic exposure (IGE) during young adulthood is associated with multiple target organs function at midlife independent of single fasting glucose (FG) measurement. They included 2,859 participants, aged 18–30 years at Y0, in the coronary artery risk development in young adults (CARDIA) Study. They found that higher intensity of glycemic exposure during young adulthood was independently associated with subclinical alterations of target organs function at midlife. Their findings highlight the importance of early screening and management of IGE in youth. Enhanced external counterpulsation (EECP) can improve carotid circulation in patients with coronary artery disease. Zhang et al. investigated the acute effect of EECP on the hemodynamic parameters in the carotid arteries before, during, and immediately after EECP in patients with hypertension, hyperlipidemia, and type 2 diabetes. They found that enhanced EECP created an acute reduction in end-diastolic velocity (EDV), peak systolic velocity (PSV), and systolic/diastolic flow velocity ratio (VS/VD) and an immediate increase in the resistance index (RI), flow rate (FR), and mean inner diameter (ID) of common carotid arteries (CCA) in patients. Additionally, EECP induced the different hemodynamic responses in in patients with different cardiovascular risk factors, which may provide theoretical guidance for making personalized plans in patients.

The opinion piece by Simko and Baka listed pleiotropic effects of ivabradine. They described that ivabradine has heart rate (HR)- and potential blood pressure (BP) reducing effects associated with target organ protection and the lack of undesirable metabolic and behavioral consequences (often seen with beta-blockers). They suggested ivabradine as a potential treatment for hypertensive patients with elevated HR, especially for those co-afflicted with metabolic disorders.

Recently, fibroblast growth factor-23 (FGF-23) has been identified as a new trigger of cardiac dysfunction. In this special issue, Vázquez-Sánchez et al. provide a review article summarizes current understandings of FGF-23 and focuses on emerging areas of FGF-23 in human cardiac events and its intracellular pathways in cardiomyocytes and fibroblasts. They described that significant changes in FGF-23 secretion induce detrimental effects on cardiac tissue and blocking these effects could be the possible new therapeutic opportunities.

Although this Research Topic has shed some insights on the relevance between cardiovascular diseases, diabetes and hypertension, we regret that we have not provided sufficient knowledge of this field. We currently re-launched the second collection of the articles with the same Research Topic. We hope that the next issue will also cover important studies as the first issue of this topic.

## Author Contributions

The author confirms being the sole contributor of this work and has approved it for publication.

## Conflict of Interest

The author declares that the research was conducted in the absence of any commercial or financial relationships that could be construed as a potential conflict of interest.

## Publisher's Note

All claims expressed in this article are solely those of the authors and do not necessarily represent those of their affiliated organizations, or those of the publisher, the editors and the reviewers. Any product that may be evaluated in this article, or claim that may be made by its manufacturer, is not guaranteed or endorsed by the publisher.

